# Transarterial Radioembolization (TARE) Global Practice Patterns: An International Survey by the Cardiovascular and Interventional Radiology Society of Europe (CIRSE)

**DOI:** 10.1007/s00270-024-03768-z

**Published:** 2024-06-24

**Authors:** Grace Keane, Marnix Lam, Arthur Braat, Rutger Bruijnen, Nathalie Kaufmann, Hugo de Jong, Maarten Smits

**Affiliations:** 1 Department of Radiology and Nuclear Medicine, University Medical Center Utrecht, 3508 GA Utrecht, The Netherlands; 2 Next Research, Contract Research Organization, Vienna, Austria; 3 Clinical Research, Cardiovascular and Interventional Radiological Society of Europe, Vienna, Austria

**Keywords:** Radioembolization, Global survey, Interventional radiology, SPECT/CT, PET/CT, Yttrium-90, Holmium-166

## Abstract

**Purpose:**

An international survey was conducted by the Cardiovascular Interventional Radiological Society of Europe (CIRSE) to evaluate radioembolization practice and capture opinions on real-world clinical and technical aspects of this therapy.

**Materials and Methods:**

A survey with 32 multiple choice questions was sent as an email to CIRSE members between November and December 2022. CIRSE group member and sister societies promoted the survey to their local members. The dataset was cleaned of duplicates and entries with missing data, and the resulting anonymized dataset was analysed. Data were presented using descriptive statistics.

**Results:**

The survey was completed by 133 sites, from 30 countries, spanning 6 continents. Most responses were from European centres (87/133, 65%), followed by centres from the Americas (22/133, 17%). Responding sites had been performing radioembolization for 10 years on average and had completed a total of 20,140 procedures over the last 5 years. Hepatocellular carcinoma treatments constituted 56% of this total, colorectal liver metastasis 17% and cholangiocarcinoma 14%. New sites had opened every year for the past 20 years, indicating the high demand for this therapy. Results showed a trend towards individualized treatment, with 79% of responders reporting use of personalized dosimetry for treatment planning and 97% reporting routine assessment of microsphere distribution post-treatment. Interventional radiologists played an important role in referrals, being present in the referring multi-disciplinary team in 91% of responding centres.

**Conclusion:**

This survey provides insight into the current state of radioembolization practice globally. The results reveal the increasing significance placed on dosimetry, evolving interventional techniques and increased technology integration.

**Graphical Abstract:**

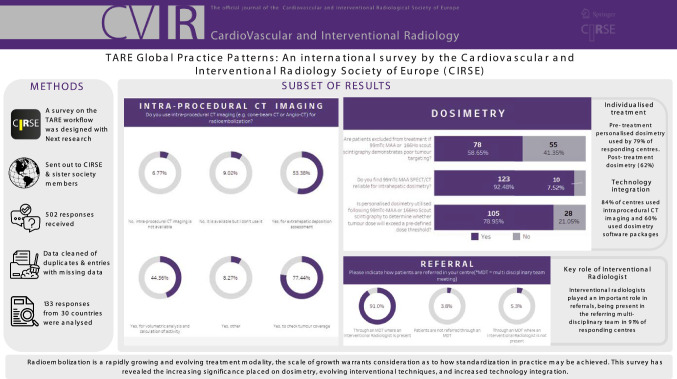

**Supplementary Information:**

The online version contains supplementary material available at 10.1007/s00270-024-03768-z.

## Introduction

Radioembolization is a complex, multi-disciplinary and multi-step intervention [1]. The treatment pathway typically begins with CT/MRI assessment [2], followed by tests to determine the clinical and biochemical status. Subsequently, a diagnostic hepatic angiography and scintigraphy using either technetium 99 m (^99m^Tc) macroaggregated albumin (MAA) or ^166^Ho scout dose is conducted, for assessment of lung shunt and intra-/extrahepatic deposition. The therapeutic procedure then takes place in a second hepatic angiography where microspheres are administered. Lastly, post-treatment imaging may be conducted to verify distribution, derive quantitative dose estimates and assess tumour response [3].

At each of the outlined steps, a multitude of decisions (regarding imaging, intent, technique, etc.) must be made, and therefore, despite being a well-established treatment option, standardization in approach is lacking [4]. Addressing variability in the field is a key challenge, especially in the context of multi-centre collaborations and clinical trials. Several studies have already been conducted to assess practice variations in Europe [5–7]. This study will expand on these previous works by obtaining a worldwide perspective that encompasses a wide variety of radioembolization centres.

The objective of this international survey was to evaluate radioembolization practice around the world and to capture a snapshot of interventional radiologist’s opinions on technical and clinical aspects of radioembolization.

## Material and Methods

### Survey Design

A survey was jointly developed by our centre and the Cardiovascular Interventional Society of Europe (CIRSE), with specific contribution from CIRSE’s Contract Research Organization Next Research. The survey consisted of 32 multiple choice questions (Table 1) in the following categories: treatment workup, treatment planning and dosimetry, intervention, follow-up and innovations. Previous surveys conducted in 2012 [6] and 2018 [5] were used as a basis when constructing the questions. Themes from the previous two iterations were maintained in the current version to allow for trending of results. Existing questions were supplemented with new topics and concepts reflecting contemporary practice, which were derived following an extensive literature review.

**Table 1:** Questions and answers as presented in the survey

Questions	Answers
What is the name of your centre?	Free text field
Where is your centre located?	City/Country
What year did your centre start performing radioembolization	Free text field
How many radioembolization procedures were performed by your centre in the following years?	2017 … 2018 … 2019 … 2020 … 2021 … 2022 (projection) …
How frequently do you encounter the following indications in your centre? (approximate percentage of total patients treated per year)	HCC … Cholangiocarcinoma …Colorectal carcinoma metastasis … Breast liver metastasis … Neuroendocrine liver metastasis … other …
How frequently do you use these microspheres for radioembolization in percent?	Y90 resin … Y90 glass … Ho166 …
What is the typical time interval between baseline diagnostic imaging (CT/MRI/other) and work up angiography?	
Do you perform liver function assessment as part of the treatment workup in some, or all of your cases?	Yes, using hepatobiliary scintigraphy (HBS)/Yes, using contrast enhanced MRI with liver specific agents/Yes, other /No
What kind of prophylactic medication do you routinely prescribe pre, during or post treatment? Where applicable, please select more than one option	Steroids/Opioids/NSAIDs/Paracetamol/Metamizole/Anti-emetics/Proton-pump inhibitor/Other
Please indicate how patients are referred in your centre(*MDT = multi-disciplinary team meeting)	Through an MDT where an Interventional Radiologist is present/Through an MDT where an Interventional Radiologist is not present/patients are not referred through an MDT
How many nights do patients normally stay in your hospital for radioembolization? Please estimate the fraction of patients per category	0 nights/1 night/ ≥ 2 nights
What is/are the main reason(s) for you to perform a scintigraphy workup procedure (99mTc-MAA or 166Ho Scout) before radioembolization? Where applicable, please select more than one option	Lung shunt assessment/Extrahepatic deposition assessment/Intrahepatic dosimetry/Other
What kind of imaging do you use to evaluate the scintigraphy workup procedure (99mTc-MAA or 166Ho Scout)? Where applicable, please select more than one option	Planar/SPECT/SPECT-CT/Other
Do you consider lung shunting a contraindication to TARE? Please select 0 for both options if you want to indicate that you do not consider lung shunting a contraindication	Yes, when the shunt is > …%/Yes, when the shunt results in a lung dose > ….Gy
How many patients (%) do you exclude due to excessive lung shunt?	
How many patients (%) receive dose reduction due to excessive lung shunting?	
Are patients excluded from treatment if 99mTc MAA or 166Ho scout scintigraphy demonstrates poor tumour targeting?	Yes/No
Is personalised dosimetry utilised following 99mTc-MAA or 166Ho Scout scintigraphy to determine whether tumour dose will exceed a pre-defined dose threshold?	Yes/No
Do you find 99mTc MAA SPECT/CT reliable for intrahepatic dosimetry?	Yes/No
What method do you use to calculate injected activity for each of the following? 90Y resin spheres/90Y glass spheres/166Ho spheres	BSA/modified BSA/MIRD single compartment/MIRD multi compartment
Do you use software for dosimetry? Where applicable, please select more than one option	Yes, MIM Sureplan/Yes, Mirada Simplicit90Y/Yes, Varian RapidSphere/Yes, Terumo QSuite/Yes, Other/No/I do not know /
Which arteries, if any, do you embolise during diagnostic angiography?	Gastroduodenal artery/Right gastric artery/Cystic artery/Other
Do you use intra-procedural CT imaging (e.g. cone-beam CT or Angio-CT) for radioembolization? Where applicable, please select more than one option	Yes, for extrahepatic deposition assessment/Yes, to check tumour coverage/Yes, for volumetric analysis and calculation of activity/Yes, other/No, intra-procedural CT imaging is not available/No, it is available but I don’t use it
What kind of microcatheter do you use for the administration of spheres? Where applicable, please select more than one option	Standard microcatheter/Anti-reflux microcatheter/Other
In what percentage of cases do you use the following sites for arterial access?	Radial …%/Femoral …%/Other …%
What is your preferred sphere administration technique in case of bilobar manifestation of tumour?	Sequential left—right radiomembolization with a time gap/Left and right hepatic artery in a single session/Whole liver (bilobar) infusion in a single session via proper hepatic artery/other
Do you use either the flexdose programme (SIRspheres) or manipulate the calibration date (TheraSphere) to adapt the number of microspheres injected? Where applicable, please select more than one option	Yes, I primarily use early week 1 TheraSphere/Yes, I primarily use late week 1 TheraSphere/Yes, I primarily use early week 2 TheraSphere/Yes, I primarily use late week 2 TheraSphere /Yes, I primarily use 1 day pre-calibration SIRsphere/Yes, I primarily use 2 days pre-calibration SIRsphere/Yes, I primarily use 3 day pre-calibration SIRsphere/Not applicable/I do not know
Do you use post-treatment imaging to visually evaluate whether the microsphere distribution is as planned? Where applicable, please select more than one option	Yes, with PET-CT/Yes, with SPECT-CT/Yes, with SPECT/Yes, with 166Ho MRI/Yes, with Other/No
Is a quantitative evaluation of post-treatment imaging performed via assessment of absorbed dose?	Yes/No
how frequently (% of all patients) do you encounter complications in radioembolization patients?	Radiation pneumonitis …%/Gastrointestinal complications …%/Pancreatic complications …%/Radioembolization induced liver disease (REILD) …%/Bile duct complications …%/Cholecystitis …%/Abscess …%/Other …%/No complication …%
Which of the following (potential) developments could improve radioembolization treatment in your practice?	New scout agents/Real-time imaging feedback on the dose distribution/Improved dose calculation methods/Improved catheter design/Same day SIRT
Are there any other emerging techniques that you think may improve radioembolization treatment in your practice?	Free text field

### Participant Recruitment

To reach an international audience and maximize participants, the survey was emailed to members of CIRSE and the Society of Interventional Radiology (SIR). CIRSE group member societies were asked to promote the survey amongst their local members. The survey was launched on the 3/11/22 and closed on the 24/12/22, and three reminder emails were sent in the interim period. Members received an email with a link to the website where the survey was hosted. Prospective responders were asked to consent to have their responses pooled, analysed and reported in a scientific publication. In the absence of consent, participants were disqualified from completing the survey.

### Data Cleaning

To allow for data extraction, the database was cleaned of duplicates, entry errors were identified and entries with missing information were removed. In case of duplicate responses, where answers differed between participants from the same centre, the response which was deemed most complete (i.e. more in-depth responses in free text fields) was selected.

### Data Analysis

Descriptive statistics were calculated for pooled responses across all sites and were used to put results into context. For multiple choice questions, where more than one response had been selected, all responses were included, which meant percentages could exceed 100%. Processing of the data was performed centrally using Tableau (ver2023.1).

## Results

### General Overview

A total of 502 responses were collected. After correcting for disqualified (7), partial (348) and duplicate (14) entries, 133 responses were used for further analysis.

**Fig. 1:**
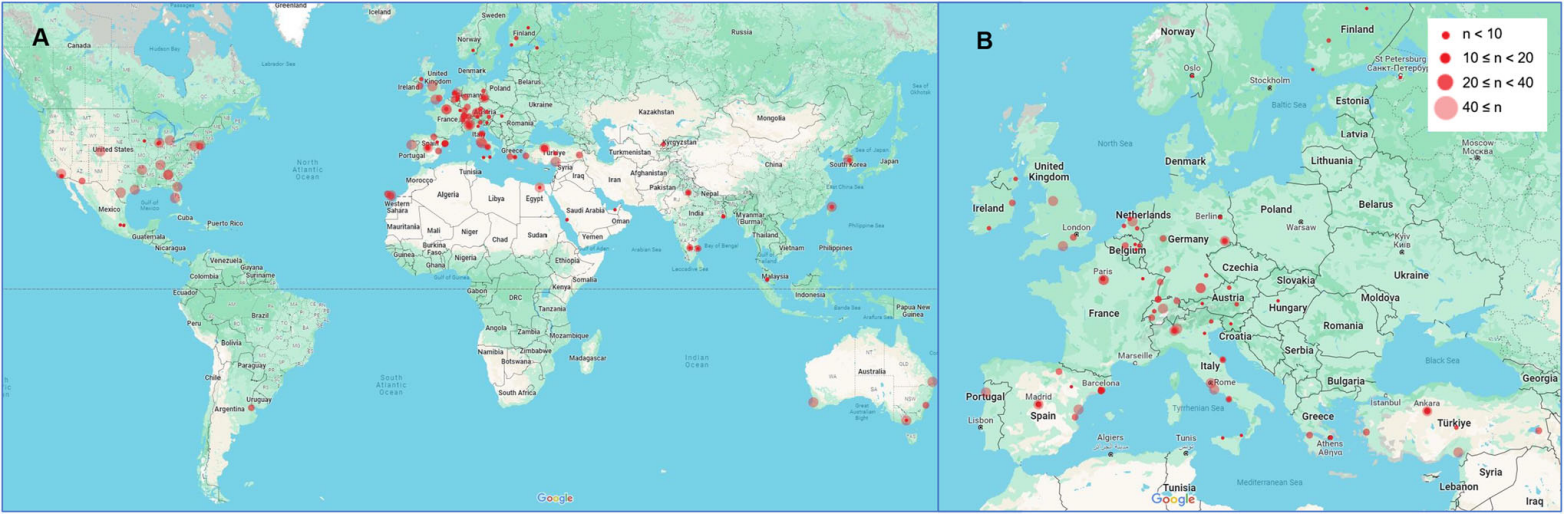
Global distribution of responding sites. **A** Geographical representation of number of radioembolization procedures per centre in 2022 globally. **B** Geographical representation of number of radioembolization procedures per centre in 2022 across Europe

The 133 responding sites were distributed across 30 countries, spanning 6 continents (Fig. 1). The majority of responses were from European centres 65%, followed by sites from the Americas 17%, Asia 13%, Oceania 4% and Africa 2%.

Cumulatively, a total of 20,140 procedures had been completed over the last 5 years across all responding sites. The total number of procedures performed per year across all sites increased by approximately 50% from 2790 in 2017 to 4165 in 2022. The average number of procedures completed by each site per year increased from 21 (range 0–230) per year in 2017 to 31 (range 0–150) in 2022. The data also revealed a substantial increase in the number of high-volume centres, defined as those performing on average at least one radioembolization procedure per week. There was triple the number of sites performing 40–60 procedures per year in 2022 compared to 2017.

Of the 20,140 procedures completed from 2017 to 2022, hepatocellular carcinoma treatments constituted 56% of this total, colorectal liver metastasis 17% and cholangiocarcinoma 14%. Lesser treated indications included breast liver metastasis 5%, neuroendocrine liver metastasis 4% and other indications 4%. Resin yttrium-90 (^90^Y)-microspheres (SIR-Spheres®, Sirtex) were used for 51% of procedures, glass ^90^Y-microspheres (TheraSphere®, Boston Scientific Corporation) 43% and holmium-166 (^166^Ho)-microspheres (QuiremSpheres®, Terumo) 5% (Table 2).

**Table 2:** Percentage split of total procedures by indications and microsphere type

Parameter	Procedures (%)
*Indications*	
Hepatocellular carcinoma	56
Metastatic colorectal carcinoma	17
Cholangiocarcinoma	14
Breast liver metastasis	5
Neuroendocrine liver metastasis	4
Other	4
*Microsphere*	
^90^Y resin	51
^90^Y glass	43
^166^Ho	5

### Pre-treatment Workup

**Fig. 2:**
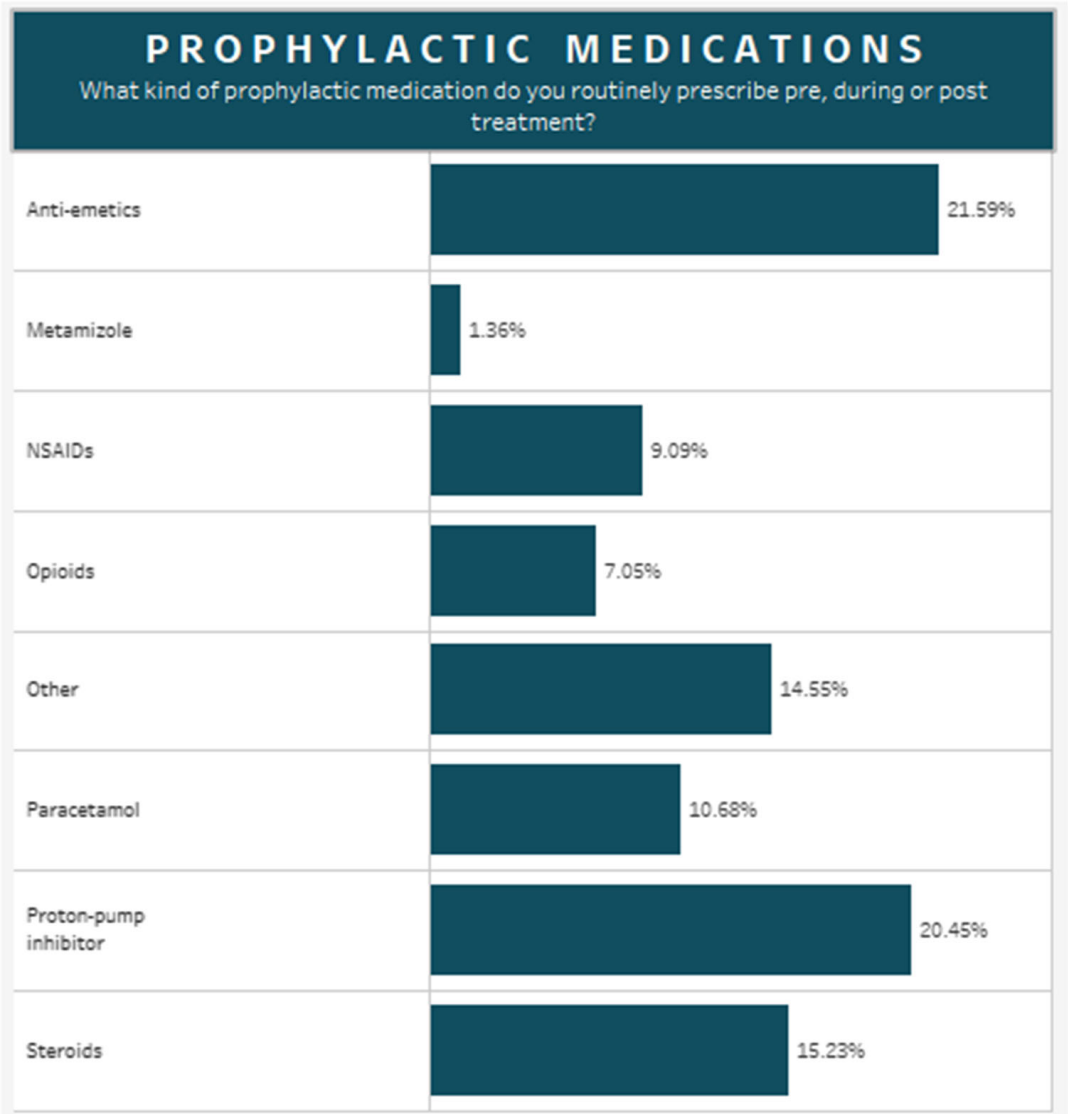
Prophylactic medications prescribed pre-, during or post-treatment

Interventional radiologists played an important role in referrals, being present in the referring multi-disciplinary team in 91% of responding centres. Prophylactic antiemetics (22%) and proton pump inhibitors (21%) were frequently prescribed before radioembolization, whereas opiates (7%) were rarely used (Fig. 2).

Image-based liver function assessment was performed by 63% of centres as part of the workup, with contrast-enhanced MRI using liver specific agents being the preferred method (26%).

### Treatment Planning and Dosimetry

**Fig. 3:**
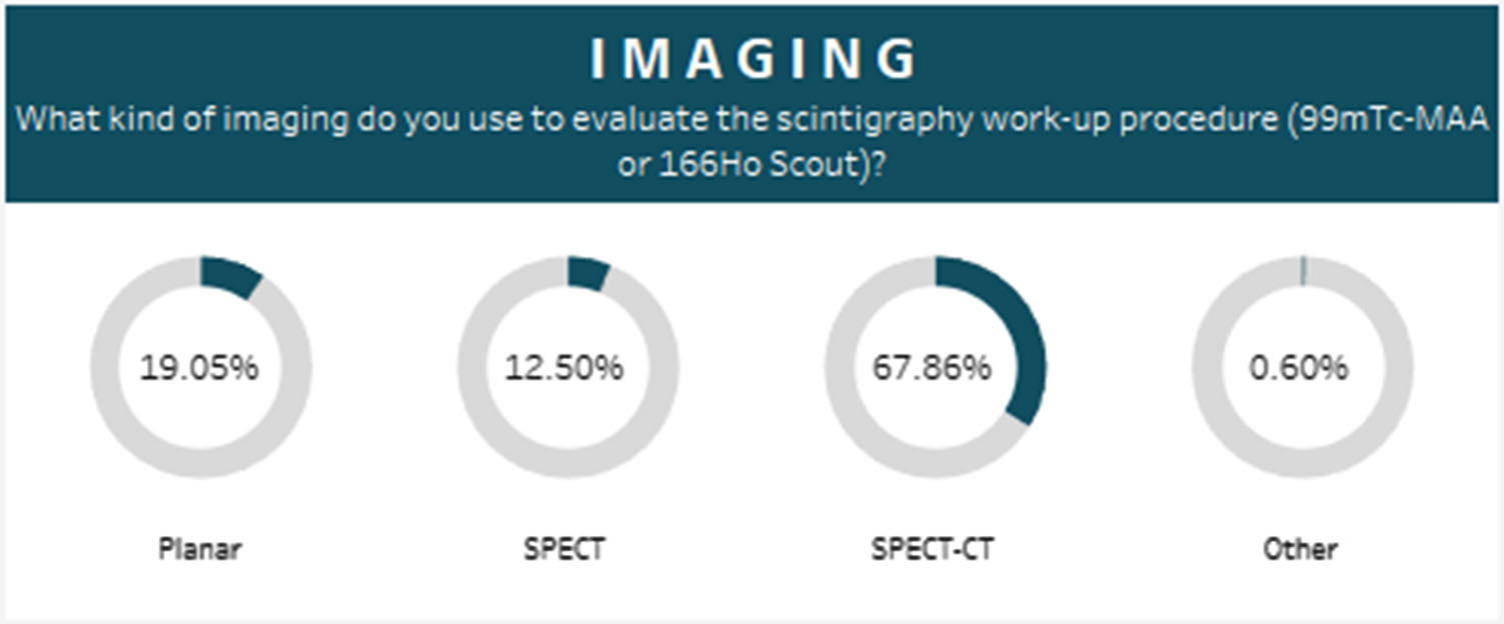
Percentage usage of imaging modalities used to evaluate the scintigraphy workup procedures (^99m^Tc MAA or ^166^Ho Scout)

The imaging modality of choice for the pre-treatment scintigraphy or ‘workup’ procedure was SPECT/CT (68%) (Fig. 3). 79% of responders utilized the workup procedure for personalized dosimetry, and specifically, to determine whether tumour dose met a pre-defined dose threshold. 59% of responders indicated they would exclude a patient from treatment if the workup procedure indicated poor tumour MAA-targeting. ^99m^Tc-MAA (i.e. the generally used scout agent prior to ^90^Y-microspheres) was considered to be reliable for intrahepatic dosimetry by a significant majority of responders (92% in favour vs. 8% against). The favoured calculation method used to determine injected activity for all radioembolization products was multi-compartment dosimetry using the MIRD (medical internal radiation dose) schema. Specifically, 51% of resin users, 52% of glass users and 61% of ^166^Ho users selected this method. Median lung shunt and lung dose values of 20.0% and 30.0 Gy, respectively, were considered a contraindication to treatment. A minority of centres (4.5%) did not consider lung shunting a contraindication.

**Fig. 4:**
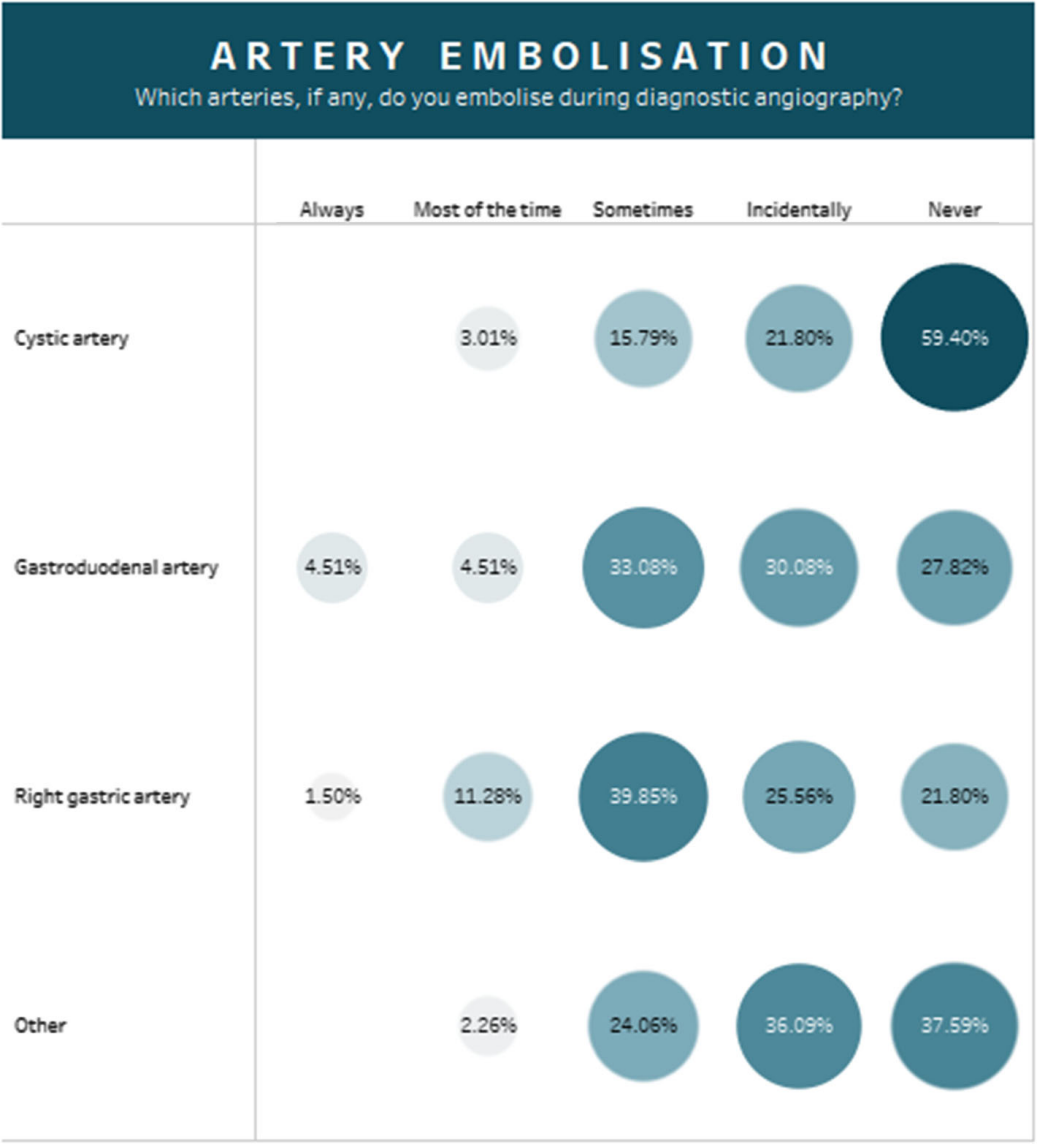
The embolization rates of arteries that may be coiled during the radioembolization workup

The majority of responders (60%) used software to support dosimetry calculations, the most commonly used package was Simplicit90Y™ (Mirada Medical) (22%), followed by SurePlan™ MRT (MIM Software Inc) (13%). Routine coil embolization of non-target vessels was rare. Overall, the right gastric artery and gastroduodenal artery were the most frequently embolized vessels (Fig. 4).

### Intervention

**Fig. 5:**
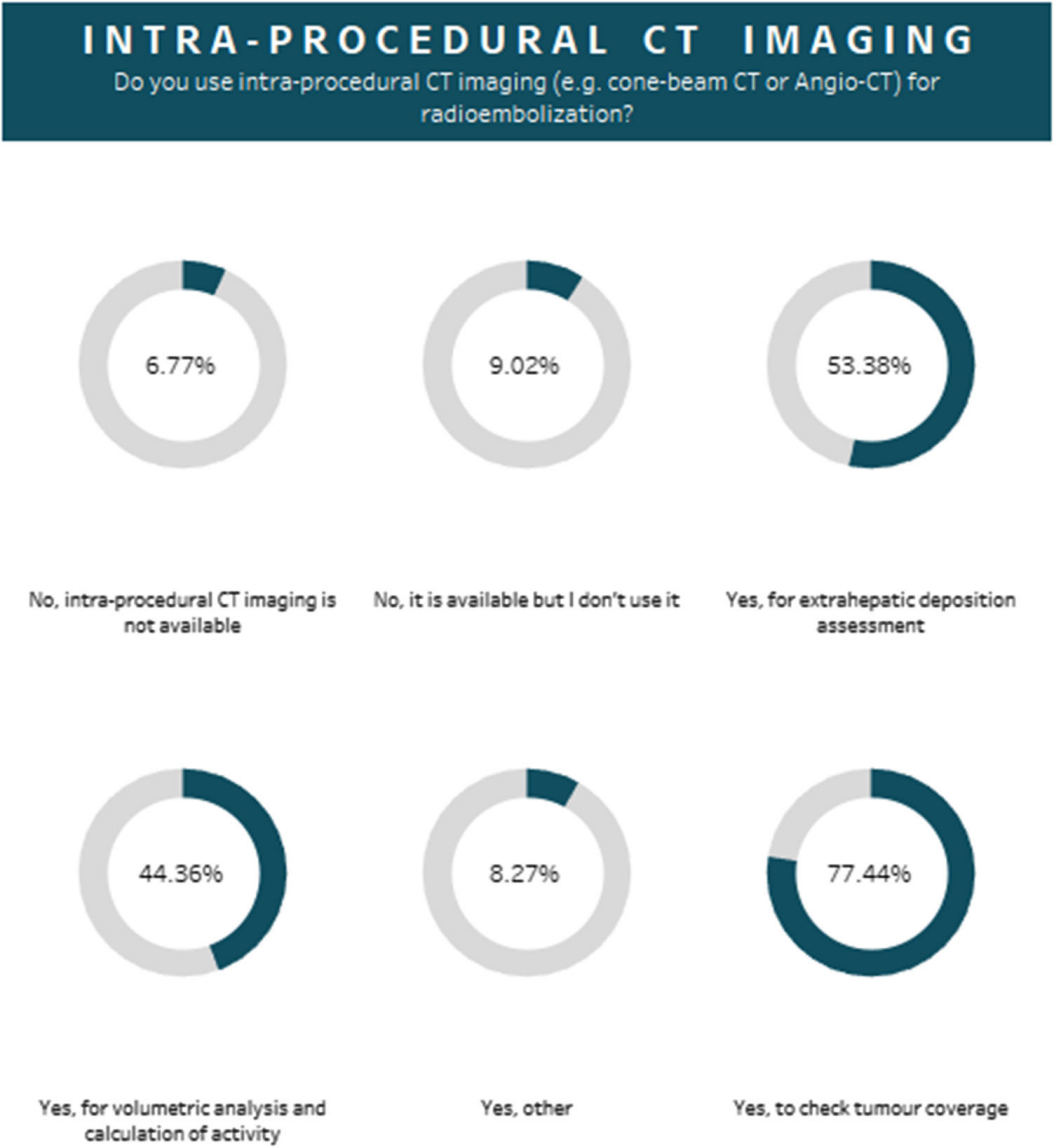
The percentage usage of intra-procedural CT imaging

A large majority of responders utilized intra-procedural CT imaging (e.g. cone-beam CT or Angio-CT) (84%), and only a small number of users did not use it (9%) or lacked access to it (7%) (Fig. 5). Most centres reported using intra-procedural CT to confirm adequate tumour coverage (77%). The majority of users considered standard catheters (as opposed to antireflux or other catheters) sufficient for administration of microspheres (87%). A femoral access route was used in 75% of cases, radial was used in 14%, and a large proportion of sites (33%) used both femoral and radial access options.

For patients with multiple tumours and/or bilobar tumour manifestation, 65% of centres opted for a sequential left/right session with an interval, and only 4% performed a whole liver (bilobar) infusion in a single session via the proper hepatic artery (Supplemental Fig. 1).

Tailoring the number of injected microspheres for different clinical indications is a method that has gained prominence in recent years [8]. Results indicated that 61% of responders did consider the number of microspheres when planning treatments. Among those using glass microspheres, the ‘late week 1’ order option was favoured by the majority (35%). For resin microspheres, the ‘1-day pre-calibration’ order option was the preferred selection (39%) (supplemental Fig. 1).

Regarding the duration of patient stay (0,1 or ≥ 2 nights) following radioembolization, the average number of patients staying 0, 1 and ≥ 2 nights was 46, 61 and 44%, respectively.

### Follow-Up

**Fig. 6:**
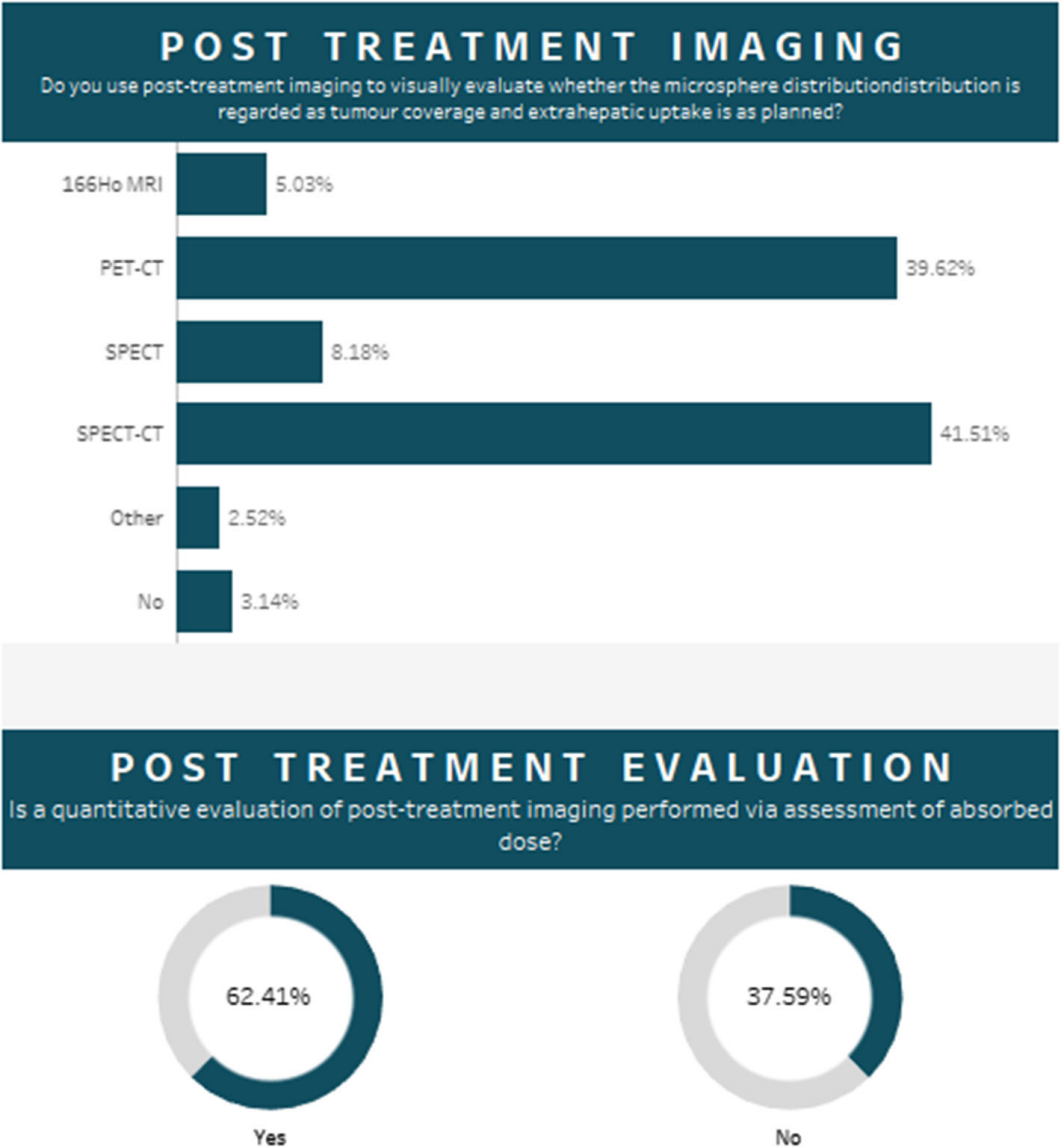
(Upper) The percentage of users that perform a quantitative evaluation of post-treatment imaging via assessment of absorbed dose. (Lower) The percentage usage of imaging modalities for post-treatment imaging. **A** The percentage usage of various administration techniques in cases of bilobar tumour manifestation. **B** The percentage usage of the calibration date options for 90Y glass and flexdose options for 90Y resin

The majority of centres performed post-treatment imaging (97%) to visually evaluate whether the microsphere distribution was as planned, SPECT/CT was the most commonly used modality for this procedure (Fig. 6). A quantitative evaluation of post-treatment imaging to determine delivered dose was performed by 62% of responders. The reported incidence of complications was low, with REILD (radioembolization induced liver disease) (6%) and gastrointestinal complications (5%) the highest reported across all centres.

### Innovations

Centres showed most enthusiasm for improved dose calculation methods, real-time imaging feedback on the dose distribution and novel scout agents, with 79, 77 and 70% of responders indicating they either agreed or strongly agreed these potential developments could improve radioembolization practice, respectively (Table 3) (Supplemental Fig. 2).

**Table 3:** Innovations

Development	Strongly Agree (%)	Agree (%)	Neutral (%)	Disagree (%)	Strongly Disagree (%)
*Which of the following developments could improve radioembolization treatment in your practice?*					
Improved catheter design	11.3	28.6	44.4	12.8	3.0
Improved dose calculation methods	33.8	45.1	13.5	2.3	5.3
New scout agents with improved predictive capability	28.6	40.6	22.6	3.8	4.5
Real-time imaging feedback on the dose distribution	37.6	38.4	17.3	3.0	3.8
Same day radioembolization	16.5	33.1	36.1	9.8	4.5

Highlighted innovations that may improve radioembolization practice varied from possible synergistic effects with immuno-oncology agents, to MRI guided administration of microspheres and temporary embolization material to redirect blood flow.

## Discussion

A comprehensive database on the real-life clinical application of radioembolization has been collated as part of this global CIRSE survey. The extent to which results accurately represent reality is dependent on the number of responders, how well the questions were understood [9], the propensity for centres involved in research to be more likely to respond, among other factors. Despite these limitations, the 133 responding centres included sites with various backgrounds and spanned the majority of geographical regions where radioembolization is conducted, giving these data a broad basis to represent the field as a whole.

Our data demonstrated an almost 50% increase in the number of patients treated with radioembolization over the last 5 years. This has persisted despite challenges associated with reimbursement [10, 11], the outcome of several negative trials [12–17] and the global COVID-19 pandemic [18–20]. European surveys in 2011 and 2018 reported data from 28 and 71 responding centres, respectively. The number of participating centres steadily increased and culminated in this work, for which 502 responses were received and 133 were analysed. Products from all three major microspheres vendors (Sirtex, Boston Scientific and Terumo) were considered. This marked a change from previous surveys that focussed solely on resin and glass microspheres. Resin remained the predominantly used microsphere.

Trends towards individualized treatment and personalized dosimetry that have been widely reported in the literature [18] were clearly evident in this survey. For example, an emerging technique gaining prominence is the trans-radial administration of microspheres, as an alternative to the standard trans-femoral route. This approach has been shown to decrease risk of entry site complications, improve post-procedural comfort levels [21, 22] and provide flexibility to treat patients with inaccessible groins/uncorrectable coagulopathy. A third of responders (33%) indicated that they now utilize both femoral and radial access routes in their centre. In addition, there was a trend towards more selective administrations to tumour-bearing segments of the liver [23, 24]. Results demonstrated reduced prophylactic coil embolization rates, which reflects the increased drive for more selective treatments.

Many of the highlighted procedural innovations are underpinned by technological developments. The precise visualization of vasculature [25] offered by intra-procedural CT imaging (e.g. cone-beam CT or Angio-CT) enables accurate detection of tumour-feeding vessels and precise targeting of lesions, facilitating the super-selective microsphere administration techniques that are increasingly observed. Results indicated high percentage usage of this modality and growth over the last 5 years; however, there were a minority (9%) of responders who indicated they have access to c-arm CT but do not use it. This potentially reveals a role for educational initiatives, to emphasize the impact of this technology on patient outcomes and the necessity for its incorporation by all centres. The increased sensitivity and specificity of ^99m^Tc-MAA SPECT/CT in the detection of extrahepatic arterial shunting [26] compared to conventional planar imaging have resulted in a shift towards this modality for the pre-treatment workup. The present results indicated that more than two-thirds of responders utilize SPECT/CT to evaluate the scintigraphy workup procedure.

While some centres still utilized semi-empirical dosimetry planning methods for radioembolization, these represented a minority (e.g. BSA dosimetry usage was < 15% for all microsphere products), and over the past five years there has been a shift in the community towards MIRD dosimetry methods that have been used in several landmark trials [27–29]. This survey demonstrated that MIRD dosimetry methods were unanimously favoured across all products, which is a change from a previous (2018) European survey [5] that indicated the partition model was "rarely" used. Consequently, it seems reasonable to assume that activity planning is now evolving along two paths: MIRD single and MIRD multi-compartment modelling. In general, dosimetry has taken on greater significance and now constitutes a central practice in the treatment workup. Our results indicated that responders frequently use dosimetric data, including projected tumour and liver doses to assist clinical decision-making.

The increasing evidence supporting personalized treatment planning [27, 30–32] has resulted in a rise in the use of dosimetry software packages. The widespread adoption of these packages will help to standardize dosimetry planning methods and improve consistency. Comparable performance that allows for reliable comparison of quantitative dose metrics between centres is essential, and harmonization strategies are the subject of ongoing research [33, 34].

A clear consensus among centres was the low incidence of complications, with average reported incidences ranging from 0 to 6%. Gastrointestinal complications and REILD were most commonly encountered, which is in line with previously reported results [5]. It should be noted that there is no clear consensus on definition of REILD and therefore responders may have interpreted what constitutes REILD differently. An example response in this survey called for ‘better predictive capabilities of who may develop REILD, as lab values can give a false impression of a normal functioning liver’. This may explain why an increasing number of centres are integrating image-based liver function tests into the pre-treatment workup, results indicating that 63% of centres now perform these tests as standard.

For this survey, there were challenges in reaching responders resulting in an irregular response rate between countries. The uneven global distribution of responses can be attributed to the lead role of CIRSE, with sister organizations being comparatively less involved. Efforts to enhance global participation should be considered for future surveys. A challenge of conducting a qualitative survey is the reliance on self-reported data; the absence of validation introduces the potential for reporting bias. Additionally, a substantial volume of data was collected and only a curated subset may be presented in this manuscript. Consequently, greater emphasis should be placed on discerning trends rather than exact numerical estimates.

This survey highlighted several prospective advancements in radioembolization which may improve practice. Notably, adaptive planning, involving intraoperative re-optimization, garnered widespread approval. Current developments including radio-opaque microspheres [35] and a novel imaging modality IXSI [36], could facilitate real-time feedback on the dose distribution, which is necessary to make adaptive planning a reality. Respondents also underscored the treatment optimization potential of personalized dosimetry (79% of responders agreed this would improve practice), suggesting a probable expansion of personalized dosimetry adoption.

## Conclusion

Radioembolization is a rapidly growing and evolving treatment modality; the scale of growth warrants consideration as to how standardization in practice may be achieved. This survey has revealed the increasing significance placed on dosimetry, evolving interventional techniques and increased technology integration. The findings underscore the value of sharing knowledge and best practice.

## Supplementary Information

Below is the link to the electronic supplementary material.

Supplementary file1 (DOCX 295 KB)

## Data Availability

The datasets used and/or analysed during the current study are available from the corresponding author on reasonable request.

## Abbreviations

**CT**: Computed tomography
**MRI**: Magnetic resonance imaging
^**99m**^**Tc****-MAA**: Technetium-99 macroaggregated albumin
**166Ho**: Holmium-166
**CIRSE**: Cardiovascular Interventional Radiological Society of Europe
**SIR**: Society of Interventional Radiology
**90Y**: Yttrium-90
**MIRD**: Medical Internal Radiation Dose
**REILD**: Radioembolization induced liver disease
