# Supplementary material for: Transarterial Radioembolization (TARE) Global Practice Patterns: An International Survey by the Cardiovascular and Interventional Radiology Society of Europe (CIRSE)
Source: Cardiovasc Intervent Radiol. 2024 Jun 24;47(9):1224–36. doi: 10.1007/s00270-024-03768-z (PMC11379766; doi:10.1007/s00270-024-03768-z)
Supplement: Supplementary file 1 — Supplementary file1 (DOCX 295 KB) [file 270_2024_3768_MOESM1_ESM.docx]

# FIGURES (SUPPLEMENTAL)

Figure 1. Results relating to microsphere administration techniques

**A**


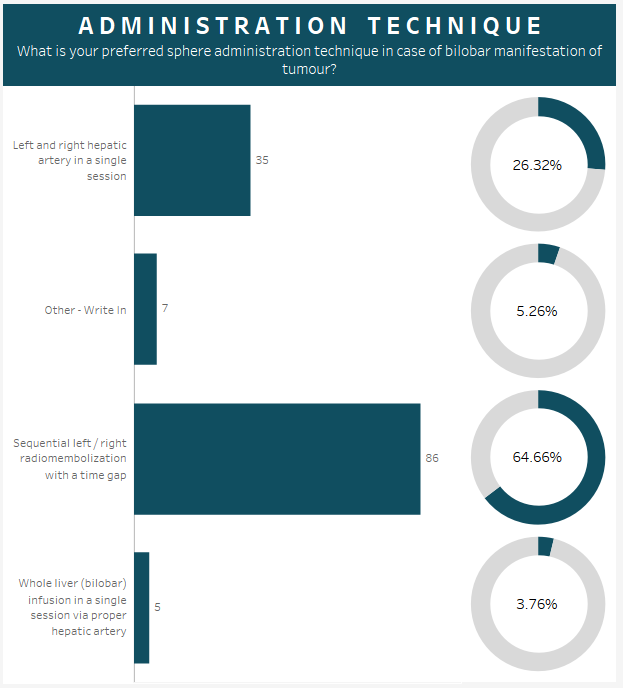

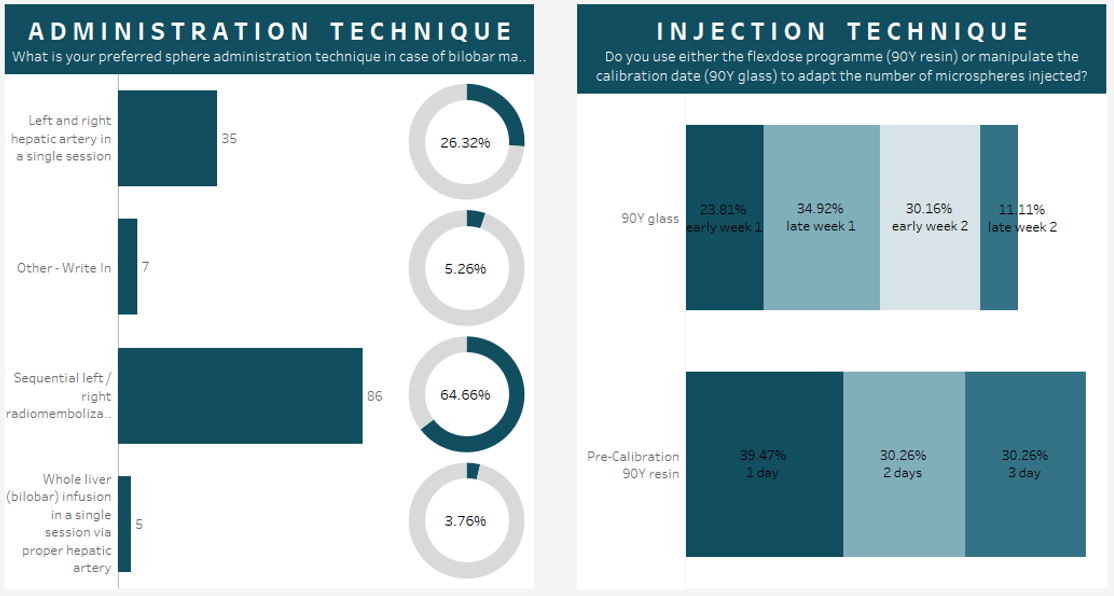


**B**

A: The percentage usage of various administration techniques in cases of bilobar tumour manifestation. B: The percentage usage of the calibration date options for 90Y glass and flexdose options for 90Y resin

Figure 2. Heat map of potential developments that could improve radioembolization treatment practice

**
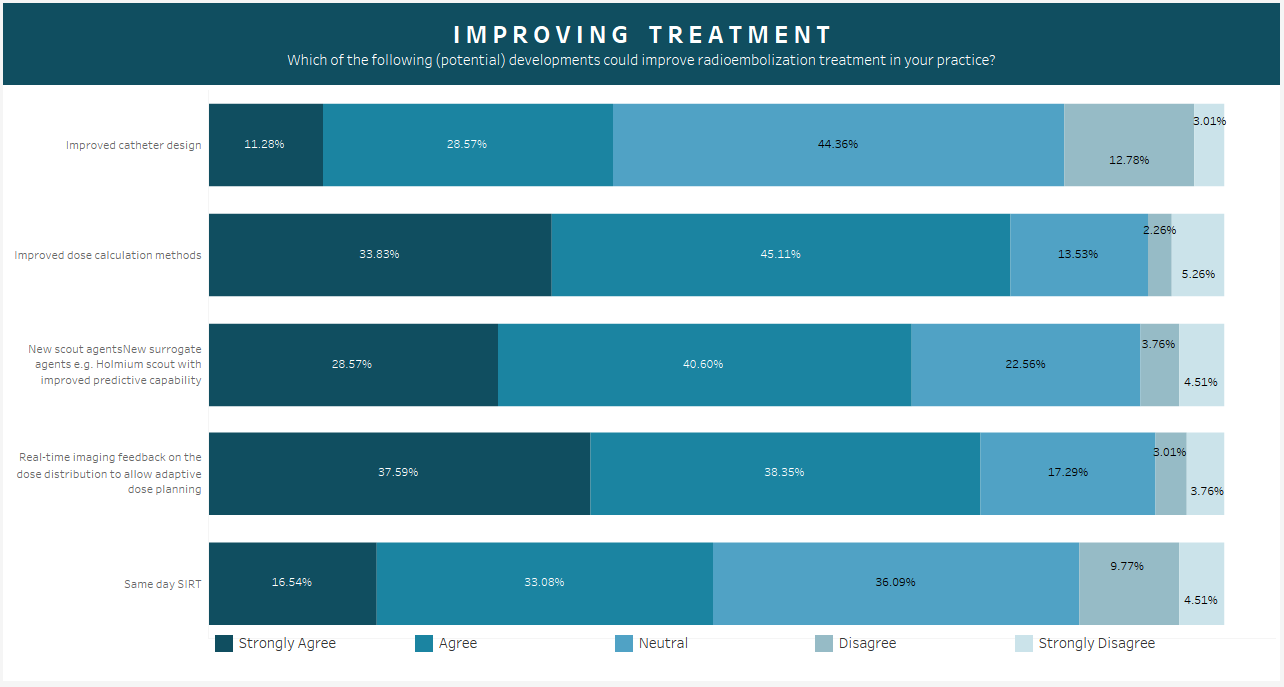
**

# TABLES (SUPPLEMENTAL)

| **Questions** | **Answers** | | **Response** |
| --- | --- | --- | --- |
| 1. What is the name of your centre? | Free text field | | N/A |
| 1. Where is your centre located? | City / Country | | N/A |
| 1. What year did your centre start performing radioembolization? | Free text field | | N/A |
| 1. How many radioembolization procedures were performed by your centre in the following years? | 2017 | | 2790 |
|  | 2018 | | 3090 |
|  | 2019 | | 3330 |
|  | 2020 | | 3145 |
|  | 2021 | | 3615 |
|  | 2022 (projection) | | 4165 |
| 1. How frequently do you encounter the following indications in your centre? (approximate percentage of total patients treated per year) | Hepatocellular carcinoma | | 56% |
|  | Metastatic colorectal carcinoma | | 17% |
|  | Cholangiocarcinoma | | 14% |
|  | Breast liver metastasis | | 5% |
|  | Neuroendocrine liver metastasis | | 4% |
|  | Other | | 4% |
| 1. How frequently do you use these microspheres for radioembolization in percent? | ^90^Y resin | | 51% |
|  | ^90^Y glass | | 43% |
|  | ^166^Ho | | 5% |
| 1. What is the typical time interval between baseline diagnostic imaging (CT / MRI / other) and work up angiography? | 0 Weeks | | 0.8% |
|  | 1 Weeks | | 18.8% |
|  | 2 Weeks | | 30.1% |
|  | 3 Weeks | | 16.5% |
|  | 4 Weeks | | 20.3% |
|  | 5 Weeks | | 2.3% |
|  | 6 Weeks | | 7.5% |
|  | 8 Weeks | | 3.0% |
|  | 12 Weeks | | 0.8% |
| 1. Do you perform liver function assessment as part of the treatment workup in some, or all of your cases? | Yes, using contrast enhanced MRI with liver specific agents | | 25.6% |
|  | Yes, using hepatobiliary scintigraphy (HBS) | | 13.5% |
|  | Yes, other | | 24.1% |
|  | No | | 36.8% |
| 1. What kind of prophylactic medication do you routinely prescribe pre, during or post treatment? Where applicable, please select more than one option. | Anti-emetics | | 21.59% |
|  | Proton-pump inhibitor | | 20.5% |
|  | Steroids | | 15.2% |
|  | Metamizole | | 1.4% |
|  | Paracetamol | | 10.7% |
|  | NSAIDs | | 9.1% |
|  | Opioids | | 7.1% |
|  | Other | | 14.6% |
| 1. Please indicate how patients are referred in your centre (*MDT = multi-disciplinary team meeting) | Through an MDT where an Interventional Radiologist is present | | 91.0% |
|  | Through an MDT where an Interventional Radiologist is not present | | 5.3% |
|  | Patients are not referred through an MDT | | 3.8% |
| 1. How many nights do patients normally stay in your hospital for radioembolization? Please estimate the fraction of patients per category | 0 nights | | 45.7% |
|  | 1 night | | 60.9% |
|  | ≥2 nights | | 43.5% |
| 1. What is/are the main reason(s) for you to perform a scintigraphy work-up procedure (99mTc-MAA or 166Ho Scout) before radioembolization? Where applicable, please select more than one option. | Lung shunt assessment | | 94.0% |
|  | Extrahepatic deposition assessment | | 76.7% |
|  | Intrahepatic dosimetry | | 74.4% |
|  | Other | | 6.0% |
| 1. What kind of imaging do you use to evaluate the scintigraphy work-up procedure (99mTc-MAA or 166Ho Scout)? Where applicable, please select more than one option | SPECT-CT | | 67.9% |
|  | Planar | | 19.1% |
|  | SPECT | | 12.5% |
|  | Other | | 0.6% |
| 1. Do you consider lung shunting a contraindication to TARE? Please select 0 for both options if you want to indicate that you do not consider lung shunting a contraindication | Yes, when the shunt is > …% | | 20.0% |
|  | Yes, when the shunt results in a lung dose > ….Gy | | 30.0Gy |
|  | No, I do not consider lung shunting a contraindication | | 4.5% |
| 1. How many patients (%) do you exclude due to excessive lung shunt? | Free text field | | 5.5% |
| 1. How many patients (%) receive dose reduction due to excessive lung shunting? | Free text field | | 7.7% |
| 1. Are patients excluded from treatment if 99mTc MAA or 166Ho scout scintigraphy demonstrates poor tumour targeting? | Yes | | 58.7% |
|  | No | | 41.4% |
| 1. Is personalised dosimetry utilised following 99mTc-MAA or 166Ho Scout scintigraphy to determine whether tumour dose will exceed a pre-defined dose threshold? | Yes | | 79.0% |
|  | No | | 21.1% |
| 1. Do you find 99mTc MAA SPECT/CT reliable for intrahepatic dosimetry? | Yes | | 92.5% |
|  | No | | 7.5% |
| 1. What method do you use to calculate injected activity for each of the following? 90Y resin spheres /  90Y glass spheres / 166Ho spheres | 90Y Resin | BSA | 14.0% |
|  |  | modified BSA | 24.3% |
|  |  | MIRD single compartment | 5.6% |
|  |  | MIRD multi compartment | 51.4% |
|  |  | Other | 4.7% |
|  | 90Y Glass | BSA | 9.2% |
|  |  | modified BSA | 17.2% |
|  |  | MIRD single compartment | 18.4% |
|  |  | MIRD multi compartment | 51.7% |
|  |  | Other | 3.5% |
|  | 166Ho | BSA | 9.1% |
|  |  | modified BSA | 9.1% |
|  |  | MIRD single compartment | 15.2% |
|  |  | MIRD multi compartment | 60.6% |
|  |  | Other | 6.1% |
| 1. Do you use software for dosimetry? Where applicable, please select more than one option | Yes, MIM Sureplan | | 13.3% |
|  | Yes, Mirada Simplicit90Y | | 21.8% |
|  | Yes, Varian RapidSphere | | 4.2% |
|  | Yes, Terumo QSuite | | 10.9% |
|  | Yes, Other | | 9.7% |
|  | No | | 22.4% |
|  | I do not know | | 17.6% |
| 1. Which arteries, if any, do you embolise during diagnostic angiography? | Gastroduodenal artery | Always | 4.5% |
|  |  | Most the time | 4.5% |
|  |  | Sometimes | 33.1% |
|  |  | Incidentally | 30.1% |
|  |  | Never | 27.8% |
|  | Right gastric artery | Always | 1.5% |
|  |  | Most the time | 11.3% |
|  |  | Sometimes | 39.9% |
|  |  | Incidentally | 25.6% |
|  |  | Never | 21.8% |
|  | Cystic artery | Always | 0.0% |
|  |  | Most the time | 3.0% |
|  |  | Sometimes | 15.8% |
|  |  | Incidentally | 21.8% |
|  |  | Never | 59.4% |
|  | Other | Always | 0.0% |
|  |  | Most the time | 2.3% |
|  |  | Sometimes | 24.1% |
|  |  | Incidentally | 36.1% |
|  |  | Never | 37.6% |
| 1. Do you use intra-procedural CT imaging (e.g. cone-beam CT or Angio-CT) for radioembolization? Where applicable, please select more than one option | Yes, to check tumour coverage | | 77.4% |
|  | Yes, for extrahepatic deposition assessment | | 53.4% |
|  | Yes, for volumetric analysis and calculation of activity | | 44.4% |
|  | Yes, other | | 8.3% |
|  | No, it is available but I don’t use it | | 9.0% |
|  | No, intra-procedural CT imaging is not available | | 6.8% |
| 1. What kind of microcatheter do you use for the administration of spheres? Where applicable, please select more than one option | Standard microcatheter | | 86.8% |
|  | Anti-reflux microcatheter | | 11.9% |
|  | Other | | 1.3% |
| 1. In what percentage of cases do you use the following sites for arterial access? (Average) | Radial …% | | 14.4% |
|  | Femoral …% | | 75.0% |
|  | Other …% | | 10.6% |
| 1. What is your preferred sphere administration technique in case of bilobar manifestation of tumour? | Sequential left - right radiomembolization with a time gap | | 64.7% |
|  | Left and right hepatic artery in a single session | | 26.3% |
|  | Whole liver (bilobar) infusion in a single session via proper hepatic artery | | 3.8% |
|  | Other | | 5.3% |
| 1. Do you use either the flexdose programme (SIRspheres) or manipulate the calibration date (TheraSphere) to adapt the number of microspheres injected? Where applicable, please select more than one option. | TheraSphere  (% of respondents using TheraSphere) | Yes, I primarily use early week 1 | 23.8% |
|  |  | Yes, I primarily use early week 2 | 34.9% |
|  |  | Yes, I primarily use late week 1 | 30.2% |
|  |  | Yes, I primarily use late week 2 | 11.1% |
|  | SIRsphere  (% of respondents using SIRsphere) | Yes, I primarily use 1 day pre-calibration | 39.5% |
|  |  | Yes, I primarily use 2 day pre-calibration | 30.3% |
|  |  | Yes, I primarily use 3 day pre-calibration | 30.3% |
|  | Not applicable (% respondents w/o positive response) | | 48.1% |
|  | I do not know (% respondents w/o positive response) | | 51.9% |
| 1. Do you use post-treatment imaging to visually evaluate whether the microsphere distribution is as planned? Where applicable, please select more than one option. | Yes, with SPECT-CT | | 41.5% |
|  | Yes, with PET-CT | | 39.6% |
|  | Yes, with SPECT | | 8.2% |
|  | Yes, with 166Ho MRI | | 5.0% |
|  | Yes, with Other | | 2.5% |
|  | No | | 3.1% |
| 1. Is a quantitative evaluation of post-treatment imaging performed via assessment of absorbed dose? | Yes | | 62.4% |
|  | No | | 37.6% |
| 1. How frequently (% of all patients) do you encounter complications in radioembolization patients? (Average % reported) | Radiation pneumonitis …% | | 3.5% |
|  | Gastrointestinal complications …% | | 5.0% |
|  | Pancreatic complications …% | | 2.1% |
|  | Radioembolization induced liver disease (REILD) …% | | 5.7% |
|  | Bile duct complications …% | | 3.9% |
|  | Cholecystitis …% | | 2.6% |
|  | Abscess …% | | 2.7% |
|  | Other …% | | 1.7% |
|  | No complication …% | | 79.8% |
| 1. Which of the following (potential) developments could improve radioembolization treatment in your practice? | New scout agents | Strongly Agree | 28.6% |
|  |  | Agree | 40.6% |
|  |  | Neutral | 22.6% |
|  |  | Disagree | 3.8% |
|  |  | Strongly Disagree | 4.5% |
|  | Real-time imaging feedback on the dose distribution | Strongly Agree | 37.6% |
|  |  | Agree | 38.4% |
|  |  | Neutral | 17.3% |
|  |  | Disagree | 3.0% |
|  |  | Strongly Disagree | 3.8% |
|  | Improved dose calculation methods | Strongly Agree | 33.8% |
|  |  | Agree | 45.1% |
|  |  | Neutral | 13.5% |
|  |  | Disagree | 2.2% |
|  |  | Strongly Disagree | 5.3% |
|  | Improved catheter design | Strongly Agree | 11.3% |
|  |  | Agree | 28.6% |
|  |  | Neutral | 44.3% |
|  |  | Disagree | 12.8% |
|  |  | Strongly Disagree | 3.0% |
|  | Same day SIRT | Strongly Agree | 16.5% |
|  |  | Agree | 33.1% |
|  |  | Neutral | 36.1% |
|  |  | Disagree | 9.8% |
|  |  | Strongly Disagree | 4.5% |
| 1. Are there any other emerging techniques that you think may improve radioembolization treatment in your practice? | Free text field | | N/A |
